# Genetic Variation in *ABCC4* and *CFTR* and Acute Pancreatitis during Treatment of Pediatric Acute Lymphoblastic Leukemia

**DOI:** 10.3390/jcm10214815

**Published:** 2021-10-20

**Authors:** Thies Bartram, Peter Schütte, Anja Möricke, Richard S. Houlston, Eva Ellinghaus, Martin Zimmermann, Anke Bergmann, Britt-Sabina Löscher, Norman Klein, Laura Hinze, Stefanie V. Junk, Michael Forster, Claus R. Bartram, Rolf Köhler, Andre Franke, Martin Schrappe, Christian P. Kratz, Gunnar Cario, Martin Stanulla

**Affiliations:** 1 Department of Pediatrics, University Hospital Schleswig-Holstein, 24105 Kiel, Germany; thyssen2@gmx.de (T.B.); a.moericke@pediatrics.uni-kiel.de (A.M.); m.schrappe@pediatrics.uni-kiel.de (M.S.); gunnar.cario@uksh.de (G.C.); 2 Department of Pediatric Hematology and Oncology, Hannover Medical School, 30625 Hannover, Germany; schuette.peter@mh-hannover.de (P.S.); Zimmermann.Martin@mh-hannover.de (M.Z.); klein.norman4@gmail.com (N.K.); hinze.laura@mh-hannover.de (L.H.); junk.stefanie@mh-hannover.de (S.V.J.); Kratz.Christian@mh-hannover.de (C.P.K.); 3 Division of Genetics and Epidemiology, Institute of Cancer Research, Sutton SM2 5NG, UK; richard.houlston@icr.ac.uk; 4 Institute of Clinical Molecular Biology, Kiel University, 24118 Kiel, Germany; e.ellinghaus@ikmb.uni-kiel.de (E.E.); b.loescher@ikmb.uni-kiel.de (B.-S.L.); m.forster@ikmb.uni-kiel.de (M.F.); a.franke@mucosa.de (A.F.); 5 Department of Human Genetics, Hannover Medical School, 30625 Hannover, Germany; bergmann.anke@mh-hannover.de; 6 Department of Human Genetics, University Hospital Heidelberg, 69120 Heidelberg, Germany; cr_bartram@med.uni-heidelberg.de (C.R.B.); Rolf.Koehler@med.uni-heidelberg.de (R.K.)

**Keywords:** acute lymphoblastic leukemia, L-asparaginase, acute pancreatitis, polymorphism, SNV, *ABCC4*, *CFTR*

## Abstract

Background: Acute pancreatitis (AP) is a serious, mechanistically not entirely resolved side effect of L-asparaginase-containing treatment for acute lymphoblastic leukemia (ALL). To find new candidate variations for AP, we conducted a genome-wide association study (GWAS). Methods: In all, 1,004,623 single-nucleotide variants (SNVs) were analyzed in 51 pediatric ALL patients with AP (cases) and 1388 patients without AP (controls). Replication used independent patients. Results: The top-ranked SNV (rs4148513) was located within the *ABCC4* gene (odds ratio (OR) 84.1; *p* = 1.04 × 10^−14^). Independent replication of our 20 top SNVs was not supportive of initial results, partly because rare variants were neither present in cases nor present in controls. However, results of combined analysis (GWAS and replication cohorts) remained significant (e.g., rs4148513; OR = 47.2; *p* = 7.31 × 10^−9^). Subsequently, we sequenced the entire *ABCC4* gene and its close relative, the cystic fibrosis associated *CFTR* gene, a strong AP candidate gene, in 48 cases and 47 controls. Six AP-associated variants in *ABCC4* and one variant in *CFTR* were detected. Replication confirmed the six *ABCC4* variants but not the *CFTR* variant. Conclusions: Genetic variation within the *ABCC4* gene was associated with AP during the treatment of ALL. No association of AP with *CFTR* was observed. Larger international studies are necessary to more conclusively assess the risk of rare clinical phenotypes.

## 1. Introduction

Acute lymphoblastic leukemia (ALL) is the most common pediatric malignancy and represents approximately 25% of cancers and 80% of all leukemias diagnosed in children and adolescents [1,2]. Contemporary treatment extends over a period of 2 to 3 years and usually consists of combination chemotherapy, which is substituted in small proportions of patients by cranial irradiation or allogeneic hematopoietic stem cell transplantation [3,4]. Timely application of therapy is important to secure optimal treatment effect and outcome but is often compromised by undesired side effects leading to treatment interruptions. Early severe side effects related to the treatment of ALL encompass a variety of specific complications, such as bacterial, viral, and fungal infections; hemostaseological problems; and side effects that can be attributed to specific drugs [5]. Examples of drug-specific toxicities observed during the treatment of ALL are methotrexate-related encephalopathy, steroid-treatment-related avascular bone necrosis, topoisomerase-II-associated secondary acute myeloid leukemia, and acute pancreatitis (AP) developing in the context of L-asparaginase (L-asp) application [6,7,8,9,10].

The mechanism of action of L-asp is the depletion of the extracellular amino acid asparagine by the hydrolysis of asparagine to aspartic acid and ammonia. The depletion results in the inhibition of protein synthesis by malignant cells, such as lymphoblasts, leading to cell death due to the inability to synthesize endogenous asparagine. L-asp used for the treatment of ALL is derived from either *Escherichia coli* (*E. coli*) (native or PEGylated L-asp) or *Erwinia chrysanthemi* [7,8,11], both being associated with AP. The mechanism of AP in association with L-asp is poorly understood. Although L-asp is believed to be the main reason for developing AP, other cytotoxic chemotherapeutics, including 6-mercaptopurine, glucocorticoids, and cytarabine, have been associated with AP, as well [12,13,14,15]. Suggested published risk factors for developing AP associated with L-asp treatment include, for example, higher age at diagnosis, acute hypertriglyceridemia, and genetic polymorphisms [11,16,17,18]. Support for an underlying genetic predisposition comes from the observation that a few applications of L-asp are sufficient to initiate AP and that there is a high probability of recurrence after re-exposure to L-asp [11]. 

So far, genetic linkage and candidate gene studies have identified several genes (e.g., *PRSS1*, *PRSS2*, *SPINK1*, *CTRC*, *CASR*, and *CFTR*) that could be associated with chronic, hereditary, and hyperlipidemic pancreatitis. Until recently, no specific loci associated with AP had been identified [11,16,19]. However, meanwhile, genome-wide association studies (GWAS) have identified single-nucleotide variants in the genes *CPA2*, *ULK2*, and *PRSS1* as being associated with L-asp-associated AP in pediatric ALL [20,21,22]. Here, we present our results from a GWAS on the etiology of AP in childhood ALL by comparing 51 patients with AP to 1388 control patients without symptoms of AP. 

## 2. Materials and Methods

### 2.1. Study Individuals 

Patients included in this study were 1 to 18 years of age and enrolled in the European AIEOP-BFM ALL 2000 multicenter clinical trial on the treatment of pediatric ALL conducted in Austria, Germany, Italy, and Switzerland [23,24]. Diagnostics and treatment in AIEOP-BFM ALL 2000 have been described previously [23,24,25,26,27]. Briefly, the AIEOP-BFM ALL 2000 patients were stratified into three branches (standard, intermediate, and high risk). Risk group stratification included minimal residual disease (MRD) analysis and required two MRD targets with sensitivities of ≤10^−4^. Standard-risk patients were MRD-negative on treatment days 33 (TP1) and 78 (TP2) and had no high-risk criteria. High-risk patients had residual disease (≥10^−3^) at TP2. MRD-intermediate-risk patients had positive MRD detection at either one or both time points but at a level of <10^−3^ at TP2. Although MRD analysis was the main stratification criterion in AIEOP-BFM ALL 2000, established high-risk parameters were also retained: patients with a poor response to prednisone or ≥5% leukemic blasts in the bone marrow on day 33 or positivity for a t(9;22) or t(4;11) or their molecular equivalents (*BCR-ABL1* or *MLL-AF4* gene fusions) were stratified into the high-risk group independent of their MRD results. Treatment details of AIEOP-BFM ALL 2000 are given in Table S1.

Diagnosis of AP was based on the presence of two of the following three clinical symptoms [28]: (1) abdominal pain consistent with acute pancreatitis (acute onset of a persistent, severe, epigastric pain often radiating to the back), (2) serum lipase activity (or amylase activity) at least three times greater than the upper limit of normal, and (3) characteristic findings of AP on abdominal computed tomography, magnetic resonance imaging, or transabdominal ultrasonography or surgical findings consistent with AP. 

### 2.2. DNA Isolation

During the course of treatment, bone marrow and/or blood samples were collected for remission evaluation at defined time points. Morphologically leukemia-cell-free samples with MRD levels of ≤10^−3^ were selected from these time points and used for DNA isolation using previously described standard techniques [26,27,29]. DNA yielded by this procedure was regarded as a germline DNA surrogate.

### 2.3. Single-Nucleotide Variant (SNV) Genotyping for Genome-Wide Screening

The GWAS was conducted in 54 childhood ALL patients with AP (cases) and 1435 patients without AP (controls). DNA was genotyped using Human1M-Duo BeadChips (Illumina, San Diego, CA, USA) containing 1,048,711 SNV markers. To avoid false positive data, 44,088 SNVs were excluded due to poor call rate (CR) (<95%) and/or deviation from Hardy–Weinberg equilibrium in the controls (*p* > 0.001). Furthermore, 37 patients (cases/controls) were excluded due to poor genotyping (CR < 95%) and cryptical relationship (IBS-distance > 0.8). Additionally, a multidimensional scaling analysis (MDS) identified 13 patients (cases/controls) with a non-European background. These subjects were also excluded from the study (Figure S1). The quality control finally resulted in a cohort size of 51 cases and 1388 controls.

Two methodological approaches were used to identify candidate SNVs for AP in this GWAS. First, only SNVs with a *p*-value smaller than 1 × 10^−7^, a minimum of one genotyping call in each group of cases and controls, and no restriction of minor allele frequency (MAF) were included. The second approach differed from the first by only including those SNVs with a MAF of more than 0.5%. Minimal evidence of an overall inflation of the test statistics due to population stratification with a moderate genomic inflation factor (approach 1: λ = 1.09; approach 2: λ = 1.10) was found (Figure S2). 

To confirm the top 20 SNVs from the GWAS, a replication analysis was conducted in an independent patient set of 54 AP cases (selected from both ALL BFM 2000 and AIEOP BFM ALL 2009 study cohorts) and 225 controls (patients with no history of AP from the ALL BFM 2000 cohort). Candidate SNVs were genotyped using the SNVlex multiplex and TaqMan technology (Applied Biosystems, Foster City, CA, USA).

### 2.4. Gene Sequencing

To fine-map *ATP-binding cassette sub-family C member 4* (*ABCC4*); 281,605 base pairs) and to evaluate the *ABCC4*-related *cystic fibrosis conductance regulator* (*CFTR*); 188,702 base pairs) gene as a candidate for AP predisposition, the two genes were completely sequenced in a cohort of 48 cases and 47 controls selected from the above-described GWAS and replication cohorts depending on the availability of sufficient amounts of non-malignant DNA. Next-generation sequencing (NGS) was conducted on a HiSeq2000 platform (Illumina) using the HaloPlex Illumina 100 kit (Agilent Technologies, Santa Clara, CA, USA) according to the manufacturer’s recommendations. The reads were mapped against the human reference genome build hg19 using BWA [30], sorted, converted to bam format, and indexed with SAMtools [31]. Local realignment around InDels and base quality score recalibration were performed with the GATK [32] according to their best practice recommendations, followed by variant calling and variant quality score recalibration. Data were analyzed using the program Integrative Genomic Viewer version 2.3.25 (www.broadinstitute.org/igv/ (accessed on 20 October 2021)) [33,34]. For identification of potential candidate SNVs, regions with a poor sequencing rate (<90%) were excluded. Follow-up SNVs in independent patients from ALL BFM 2000 and AIEOP BFM ALL 2009 with available non-malignant DNA (most of which were part of the initial GWAS and replication cohorts) were analyzed by a Sanger sequencing using an automated fluorescent sequencer (Applied Biosystems 3730xl DNA Analyzer). All data referring to chromosomal positions were based on GRCh37/hg19 assembly.

### 2.5. Plotting

**Figure 1:**
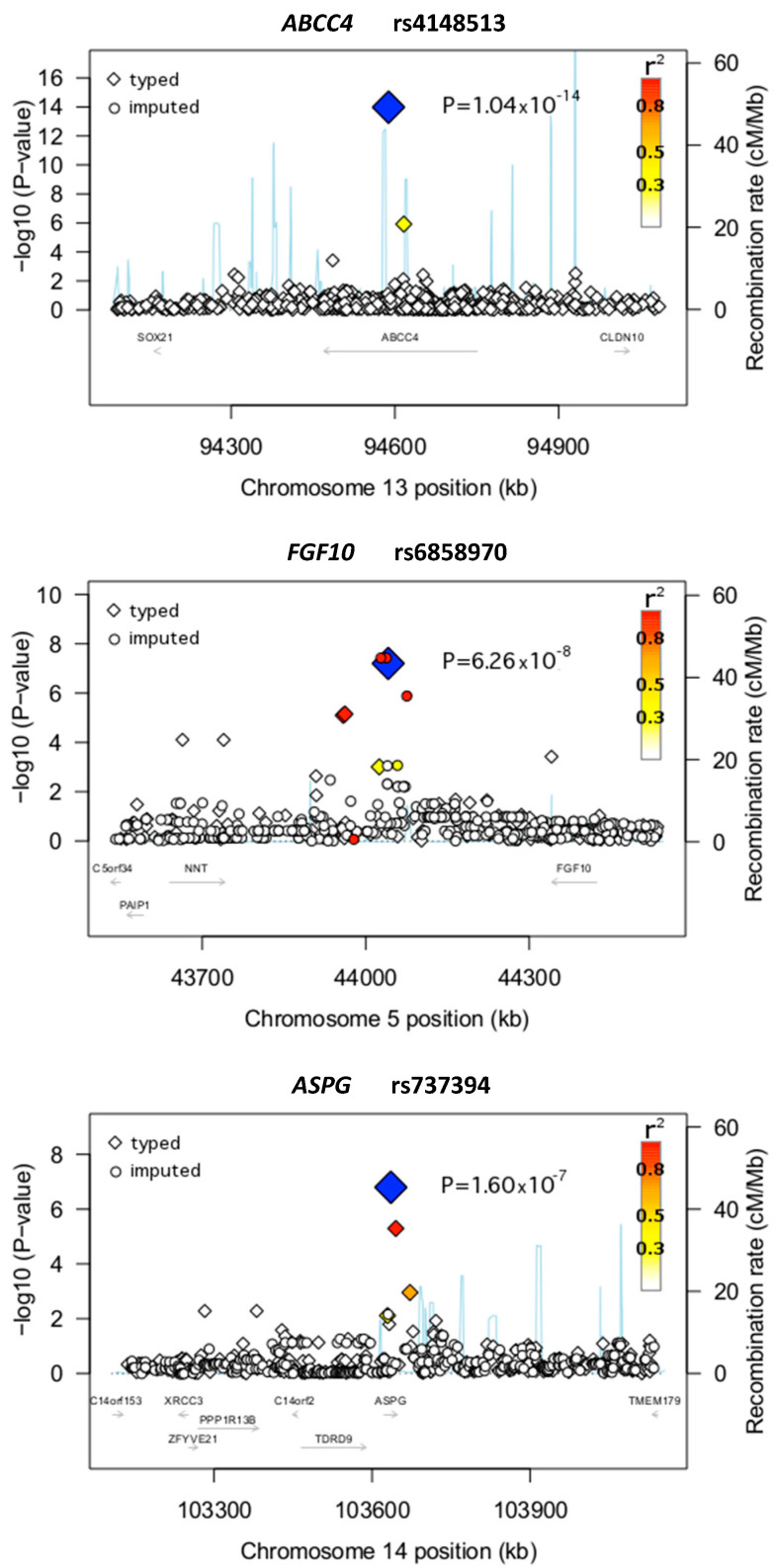
Regional plots of the loci *ABCC4*, *FGF10*, and *ASPG*. Plots of the negative decadic logarithm of the combined *p*-values obtained in the GWAS are shown. The data were imputed with CEU haplotypes generated by the 1000 Genomes Project (August 2010 release) as a reference. A window of ±500 kb around the lead SNVs (blue solid diamonds) is indicated. The magnitude of the linkage disequilibrium with the central SNV measured by r^2^ is reflected by the color of each SNV symbol (color coding: see the upper-right corner of the plot). Recombination activity (in centimorgans (cM) per Mb) is depicted by a blue line. Positions are given as NCBI’s build coordinates.

Regional association plots were created for the GWAS SNVs using a modified version of deBakker’s R script (Figure 1, Figures S3 and S4) by using GWAS SNVs as well as imputed SNVs (if possible). The imputation was done using gPLINK version 2.050 in combination with PLINK v1.07 (www.pngu.mgh.harvard.edu/purcell/plink/ (accessed on 20 October 2021)) [35]. For this purpose, genotypes of autosomal SNVs based on data of 1000 genomes were used. As an input for imputation, only SNVs from the GWAS that passed the above-mentioned quality controls were included.

### 2.6. Statistical Analyses

Associations between patient characteristics were evaluated using Fisher’s exact or χ^2^-tests. The GWAS was assessed using gPLINK. Associations of variations detected by NGS and the replication analyses in the respective cohorts used unconditional logistic regression analysis or Fisher’s exact test. Quality control and identity-by-state analysis of the GWAS data was evaluated by gPLINK and R statistics version 2.15.1 (www.r-project.org (accessed on 20 October 2021)). To estimate the European ancestry of the GWAS cohort, the multidimensional scaling analysis was evaluated using R statistics with HapMap CEU, YRI, and JRT/CHB cohorts as reference ancestral populations. Computations were performed using IBM SPSS statistics (IBM Corp., Version 21.0.0, Armonk, NY, USA) and R statistics.

## 3. Results

### 3.1. GWAS-Based Identification and Replication of Genomic SNVs Associated with AP 

In our GWAS cohort, the incidence of AP was 3.6%, which was in the range of the reported incidence of childhood-ALL-therapy-associated pancreatitis (0.7–18%) [6,7,10]. One previously described clinical risk factor associated with AP development during the treatment of childhood ALL is higher patient age, which was also observed in our analysis (Table 1) [7,11,17,18]. No significant associations of AP with the treatment risk group were detected (Table 1).

As mentioned above, our study used two methodological approaches to detect potential associations for developing AP. In the first approach, six SNVs fulfilled the predefined criteria for significance (Table 1; Figure 1 and Figure S3). An intronic SNV in the *ABCC4* gene (rs4148513) demonstrated the strongest association with AP (*p* = 1.04 × 10^−14^; OR = 84.09) (Figure 1; Table 2). Of interest, besides rs4148513, another SNV in *ABCC4* was independently and highly associated with AP in the GWAS (rs4148500; *p* = 7.23 × 10^−6^) (Table 2). Other genes with significant associations in the first GWAS approach included *SEMA3D*, *C15orf41*, *COG5*, *ST7*, and *UPF1*.

In the second approach, 13 highly significant SNVs were identified (Table 3; Figure 1 and Figure S4). The SNV with the strongest association (rs6858970) was detected close to the *fibroblast growth factor 10* (*FGF10*) gene (*p* = 6.26 × 10^−8^; OR = 8.61) (Figure 1; Table 3). Another highly associated SNV in this approach was rs737394 (*p* = 1.59 × 10^−7^; OR = 3.19), an SNV located on an intronic region of the *asparaginase homolog* (*S. cerevisiae*) (*ASPG*) gene (Figure 1; Table 3)). Other SNVs identified by the second approach were located on or in the vicinity of genes associated with mechanisms and pathways such as cell growth, cell differentiation, and cell death (Table 3).

In total, 20 SNVs were detected by our two GWAS approaches. Six of them were found to be located in intergenic regions, whereas 14 SNVs were discovered directly on a gene (Table 2, Table 3, and Table S2). All of these 20 SNVs were genotyped in additional independent patient samples (54 cases with AP and 225 controls without AP). However, none of the 20 SNVs yielded significant results in replication experiments (Table 2 and Table 3). The most significant SNV of the GWAS from the first approach (rs4148513) was neither detected in an additional case nor detected in an additional control individual. 

### 3.2. SNVs from Candidate Gene Studies and GWAS

We investigated all SNVs present on our array platform that were located on or in the vicinity of those genes previously associated with changes in susceptibility to pancreatitis, including *CFTR*, *CTRC*, *PRSS2*, *SPINK1*, *CASR*, and the recently reported variants in *AP-associated carboxypeptidase A2*-encoding gene *CPA2*, in *unc-51 like autophagy activating kinase 2*-encoding gene *ULK2*, and in *serine protease 1*-encoding gene *PRSS1* [20,21,22] but could not replicate any of the previously described significant associations (Table S3).

### 3.3. Fine-Mapping of Potential AP-Associated Variants by Sequencing the ABCC4 and CFTR Genes

Out of the 20 SNVs, the 2 with the highest significance in the GWAS approach were located on the *ABCC4* gene. *ABCC4* is a member of the superfamily of ATP-binding cassette (ABC) transporters, which also includes *CFTR*. Since patients with cystic fibrosis are prone to developing pancreatic problems, including pancreatitis, *CFTR* is a relevant candidate gene for pancreatitis in non-CF patients. The relationship to *ABCC4* as well as the candidate gene status of *CFTR* for AP led us to include both genes, *ABCC4* and *CFTR*, in a targeted NGS-based sequencing approach applied to 48 cases with AP and 47 controls without AP. In total, seven SNVs were significantly associated with AP according to the significance criteria mentioned above (see Section 2; Table 4). All NGS-based SNVs with significant associations were confirmed by Sanger sequencing. Six of the seven variants were located on the *ABCC4* gene and only one on the *CFTR* gene. One of the most significantly associated variants was the insertion rs34839857 (*p* = 1.0 × 10^−2^) in *ABCC4*, with 21 alleles present in the case group and 7 in controls. Results by genotype for the seven SNVs are given in Table S4 (Table S5 demonstrates the below-described replication and Table S6 the joint analysis of both cohorts used in fine-mapping analysis). Linkage disequilibrium (LD) analyses are demonstrated in Tables S7 and S8. The top candidate SNV from the GWAS showed no LD with any of the newly NGS identified *ABCC4* SNVs. 

## 4. Discussion

It is assumed that chemotherapeutic drugs (mainly L-asp) are the main trigger for AP in the therapeutic course of childhood ALL [6,7,8,9,10,11,13,36]. In our analyses, we were able to confirm higher age as a previously published risk factor for developing AP associated with L-asp treatment (Table 1) [7,11,17,18]. In contrast, we did not detect significant associations of AP with the treatment risk group. Several studies have analyzed the effect of risk stratification for ALL treatment as a risk factor for AP with controversial results [36,37,38]. The observed positive associations are most likely explained by higher doses of L-asp being applied in high-risk patients [36,37]. In comparison to standard- and intermediate-risk patients, our high-risk patients also received higher cumulative doses of L-asp (Table S1). Despite higher frequencies of AP in high-risk patients observed in our study, no significant differences could be detected. This is most likely due to a lack of power in our relatively small sample set. 

In addition to demographic or clinical risk factors, there is evidence of genetic factors contributing to the pathophysiology of AP as a severe treatment complication. In our first GWAS approach with no restrictions on MAF, the strongest association was observed for an SNV located on the *ABCC4* gene. *ABCC4* belongs to the ABC transporter superfamily, which mediates the efflux of drugs and plays an important role in the development of drug resistance. *ABCC4* itself is known to mediate the transport of different chemotherapeutic drugs out of the cell (e.g., 6-mercaptopurine and methotrexate) [39,40,41,42]. Therefore, variability in *ABCC4* activity may affect pharmacokinetics of *ABCC4* transport substrates and consequently modulate drug effects. Of importance in the context of our findings, *ABCC4* is highly expressed in the pancreas [39,43]. In addition, in a recent study using a rat model to study AP, Ventimiglia and colleagues described a protective role of atrial natriuretic factor (ANF) mediated by cAMP extrusion through *ABCC4* and suggested that the regulation of *ABCC4* by ANF could be relevant to maintaining pancreatic acinar cell homeostasis [44]. 

The top-ranked SNV in our second GWAS approach, which included SNVs with a MAF of more than 0.5%, was located in the vicinity of *FGF10*, a gene belonging to the fibroblast growth factor family. Members of this group take part in the regulation of cell growth and cell differentiation. In addition, the *FGF*-family is suspected to be involved in pancreatic diseases such as pancreatic cancer, chronic pancreatitis, and acute pancreatitis [45,46,47]. The *FGF10* gene itself is required for the normal development of the pancreas [47,48]. In a publication of Ishiwata et al., the authors proposed that *FGF10* together with *FGF7* may contribute to the regeneration and differentiation of acinar cells and the angiogenesis of AP [49]. However, despite *FGF10* being a plausible candidate for a role in the pathophysiology of AP, our replication analysis did not support the initial findings.

As mentioned above, one of the most serious adverse events of L-asp treatment is AP. L-asp catalyzes the hydrolysis of asparagine into aspartate and ammonia. The human genome encodes at least three enzymes that can catalyze this reaction, *asparaginase homolog (S. cerevisiae)* (*ASPG*), *aspartylglucosaminidase* (*AGA*), and *asparaginase like 1* (*ASRGL1*) [50]. Of interest, one SNV selected for further follow-up after our initial GWAS screen was located on the gene *ASPG*. This little studied gene has sequence similarity at the N-terminal domain with the *E. coli* types I and II asparaginase [51,52]. It has also been shown that HEK293 cells exhibit asparaginase activity when they are transfected with the cDNA of *ASPG* [53]. Although purely hypothetical, this initial finding, which did not hold in replication analysis, may justify some follow-up investigations of *ASPG* activity in the context of AP development.

We investigated all SNVs present on the GWAS SNV array that were located on or in the vicinity of the genes known to be associated with changes in susceptibility to pancreatitis, including *CFTR*, *CTRC*, *PRSS2*, *SPINK1*, and *CASR*, but did not find any significant association. Therefore, these previously described candidate genes for chronic pancreatitis may not play distinct roles in AP. However, we also failed to detect any association with *CPA2*, *ULK2*, and *PSSR1*, three recently reported AP-associated genes [20,21,22] (Table S3). Regarding this, our analyses may have been hampered by suboptimal SNV coverage of these candidates on our array (e.g., *CFTR*: 140 SNVs in or ±50 kb up and downstream of the gene) and the fact that hardly any of the few well-known SNVs previously associated with pancreatitis, including the top *CPA2* SNV, were actually present on our platform. LD information on this *CPA2* variant (rs199695765) could not be obtained, probably due to its rareness, so there can be no conclusions drawn from *CPA2* variants present on O1MQR. However, one of the recently published *PRSS1* variants was genotyped, showing no association to the AP phenotype (as shown in Table S3). The other published variant is not present on O1MQR but in perfect LD with the first one. The previously published *ULK2* variant rs281366 was also not genotyped on O1MQR but Table S3 lists several SNVs, for example rs205111, rs9895806, and rs9914674, that are highly linked to the published variant. In summary, our GWAS setting could not replicate the associations of rare or common SNVs to the phenotype of AP that was identified in previously published GWA studies.

Replication of the 20 top candidate SNVs from our GWAS was, unfortunately, not successful. The reasons are manifold, including the fact that our GWAS included rare variants with a low MAF. GWAS analyses often begin by discarding all genotypes for SNVs with a MAF of less than 10%, which results in an enormous loss of data. Low-MAF SNVs are associated with technical and statistical problems, such as lower genotyping rates and inflated false-positive results [53]. The decision to include rare alleles in our analyses was based on the hypothesis that AP is a rare clinical phenotype and may be associated with rare SNVs. From a methodological perspective on GWAS analyses, our practical approach is supported by investigations demonstrating nominally significant results occurring significantly less often than expected for low-MAF SNVs, resulting in a conservative bias [54,55]. However, despite positive arguments to include SNVs of low MAF, our replication cohorts may have been virtually too small to reliably detect enough cases carrying rare variants. For example, the highest-ranked SNV in our GWAS (rs4148513) occurred in three cases and one control only and was not detected in a single individual of the entire validation cohort. Nevertheless, combined data from our GWAS and the validation cohorts still demonstrated strong associations of initially identified candidate variations with AP, supporting the assumption that the initially detected SNVs might truly play a role in the development of AP. 

Lending additional support to our findings from initial experiments, we conducted fine-mapping of *ABCC4* by sequencing the entire gene. *ABCC4* was chosen because of our GWAS findings and its simultaneous candidate status based on biological function (see above). As a second candidate gene for pancreatitis, *CFTR* was chosen for sequencing [56,57,58]. *CFTR* also belongs to the ABC transporter superfamily and plays a role in water and salt transport at the plasma membrane of epithelial cells. Mutations in *CFTR* lead to cystic fibrosis (CF) commonly affecting the lungs, liver, intestine, and pancreas [59]. Moreover, variants within *CFTR* associated with pancreatitis were found in patients without additional symptoms of CF [19,60]. *CFTR* as a genetic risk factor for AP and chronic pancreatitis was linked with trypsin activation and survival in pancreatitis patients [60,61]. Of particular interest, in replication analysis of seven candidate SNVs in *ABCC4* and *CFTR* detected through NGS, all six *ABCC4* variants demonstrated similar effects regarding point estimates while the *CFTR* SNV did not. Its consistent behavior in our different analytical approaches, including genotype analysis, implies that *ABCC4* might truly be associated with AP. 

To conclude, for the first time, we were able to associate germline genetic variation in *ABCC4* with the risk of AP during treatment for childhood ALL. Our results demonstrate that *ABCC4* was consistently related to AP in GWAS as well as in fine-mapping analyses by NGS, supporting a true role of *ABCC4* in the development of AP. However, our study on a rare phenotype in a rare disease also clearly demonstrates that international joint efforts are needed to more reliably assess genetic risk factors for AP and other rare toxicities observed in childhood ALL by using larger pooled patient cohorts.

## Figures and Tables

**Table 1:** Clinical characteristics of 1439 patients with ALL from trial AIEOP-BFM ALL 2000 (GWAS cohort) according to the acute pancreatitis (AP) status.

	Patients with AP	Patients without AP	*p*-Value ^d^
	(n = 51) n (%)	(n = 1388) n (%)	
Gender			
Male	32 (62.7)	792 (57.1)	
Female	19 (37.3)	596 (42.9)	0.42
Age at diagnosis (years)			
1–6	19 (37.2)	755 (54.4)	
6 to <10	9 (17.6)	248 (17.9)	
≥10	23 (45.1)	385 (27.7)	0.02
Initial WBC ^a^ (µL)			
<10,000	21 (41.2)	579 (41.7)	
10,000–20,000	11 (21.6)	220 (15.9)	
20,000–50,000	7 (13.7)	242 (17.4)	
≥50,000	12 (23.5)	347 (25.0)	0.70
Immunophenotype			
B	37 (72.5)	1065 (76.7)	
T	14 (27.5)	304 (21.9)	0.56
Other/unknown	0	19 (1.4)	
Treatment risk group			
Standard	13 (25.5)	418 (30.1)	
Intermediate	25 (49.0)	723 (52.1)	
High	13 (25.5)	246 (17.7)	0.37
Unknown	0	1 (0.1)	
*ETV6/RUNX1*			
Neg	45 (88.2)	1149 (82.8)	
Pos	1 (2.0)	94 (6.8)	0.25
Unknown	5 (9.8)	145 (10.4)	
*BCR/ABL*			
Neg	50 (98.0)	1304 (93.9)	
Pos	1 (2.0)	24 (1.7)	0.26
Unknown	0	60 (4.3)	
*MLL* rearrangement			
Neg	46 (90.2)	1213 (87.4)	
Pos	0	4 (0.3)	0.85
Unknown	5 (9.8)	171 (12.3)	
Prednisone response ^c^			
Good	43 (84.3)	1201 (86.5)	
Poor	8 (15.7)	174 (12.5)	0.70
Unknown	0	13 (0.9)	
DNA index ^b^			
<1.16	24 (47.1)	651 (46.9)	
≥1.16	2 (7.7)	158 (11.4)	0.21
Unknown	25 (49.0)	579 (41.7)	
Timepoint of AP diagnosis ^e,f^			
Induction/consolidation (weeks 1–10)	30 (58.8)	--	
CNS-directed therapy (weeks 12–20)	5 (9.8)	--	
Re-induction (weeks 22–28)	16 (31.4)	--	--

^a^ WBC, white blood cell count at diagnosis. ^b^ Ratio of DNA content of leukemic G^0^/G^1^ cells to normal diploid lymphocytes. ^c^ Good: <1000 leukemic blood blasts/µL on treatment day 8; poor: ≥1000 µL^−1^. ^d^ χ^2^—or Fisher’s exact test. ^e^ L-asparaginase application during induction/consolidation and re-induction. ^f^ Only a few patients (<10%) developed AP after the first dose of L-asp. The majority of cases (>80%) were of severe phenotype [28], and L-asp activity levels were not available for most of them.

**Table 2:** Top SNVs associated with AP identified by genome-wide association analysis and replicated by Sanger sequencing: approach 1.

Chr. Position (bp)	dbSNP ID	Nearby Genes (Relative Position)	A1/A2	Genome-Wide Association Study			Replication			Combined Analysis GWAS + Replication		
				1388 Controls			225 Controls			1613 Controls		
				51 Cases			54 Cases			105 Cases		
				AF_A1_ Contr.	OR		AF_A1_ Contr.	OR		AF_A1_ Contr.	OR	
				AF_A1_ Cases	(95% CI)	*p*-Value ^a^	AF_A1_ Cases	(95% CI)	*p*-Value ^a^	AF_A1_ Cases	(95% CI)	*p*-Value ^a^
13	rs4148513	*ABCC4*	A/G	0.0004	84.09		0	NA		0.0003	47.20	
95790353		(within gene)		0.0294	(8.67–815.6)	1.04 × 10^−14^	0		NA	0.0144	(4.89–455.70)	7.31 × 10^−9^
7	rs17160216	*SEMA3D*	G/A	0.0007	42.03		0.0022	NA		0.0009	15.72	
85465201		(±714 kb)		0.0294	(6.95–254.4)	8.28 × 10^−12^	0		0.63	0.0144	(3.15–78.38)	6.29 × 10^−6^
15	rs698457	*C15orf41*	G/T	0.0032	15.85		0.0022	4.26		0.0031	9.55	
36372995		(±499 kb)		0.0490	(5.21–48.17)	6.75 × 10^−11^	0.0094	(0.26–68.62)	0.27	0.0289	(3.44–26.53)	1.27 × 10^−7^
7	rs6963190	*COG5*	T/C	0.0022	18.84		0.0022	4.28		0.0022	11.33	
106850181		(within gene)		0.0392	(5.23–67.85)	4.21 × 10^−10^	0.0094	(0.26–68.92)	0.26	0.0240	(3.56–36.00)	2.22 × 10^−7^
7	rs7804397	*ST7*	T/G	0.0011	28.01		0	NA		0.0009	15.72	
116857547		(within gene)		0.0294	(5.58–140.50)	7.23 × 10^−10^	0		NA	0.0144	(3.15–78.38)	6.29 × 10^−6^
19	rs2238652	*UPF1*	T/C	0.0011	28.01		0.0022	4.20		0.0012	15.64	
18942559		(within gene)		0.0294	(5.58–140.50)	7.23 × 10^−10^	0.0093	(0.26–67.63)	0.27	0.0191	(3.88–62.99)	2.12 × 10^−7^
13	rs4148500	*ABCC4*	T/C	0.0040	10.26		0.0067	NA		0.0043	4.50	
95818288		(within gene)		0.0392	(3.21- 32.79)	7.23 × 10^−6^	0		0.40	0.0192	(1.47–13.79)	3.94 × 10^−3^

Abbreviations: A1, minor allele; A2, major allele; AF, allele frequency; Chr., chromosome; CI, confidence interval; NA, not analyzed; OR, odds ratio. ^a^ Allele-based χ^2^-test (1 degree of freedom); chromosomal location is based on hg19.

**Table 3:** Top SNVs associated with AP identified by genome-wide association analysis and replicated by Sanger sequencing: approach 2.

Chr. Position (bp)	dbSNP ID	Nearby Genes (Relative Position)	A1/A2	Genome-Wide Association Study			Replication			Combined Analysis GWAS + Replication		
				1388 Controls			225 Controls			1613 Controls		
				51 Cases			54 Cases			105 Cases		
				AF_A1_ Contr.	OR		AF_A1_ Contr.	OR		AF_A1_ Contr.	OR	
				AF_A1_ Cases	(95% CI)	*p*-Value ^a^	AF_A1_ Cases	(95% CI)	*p*-Value ^a^	AF_A1_ Cases	(95% CI)	*p*-Value ^a^
5	rs6858970	*FGF10*	T/G	0.0072	8.61		0.0111	0.85		0.0078	4.46	
44005497		(±300 kb)		0.0588	(3.38–21.93)	6.26 × 10^−8^	0.0094	(0.10–7.33)	0.88	0.0337	(1.90–10.43)	1.64 × 10^−4^
1	rs12402476	*SPAG17*	A/G	0.0072	8.61		0.0089	1.06		0.0074	4.64	
118847717		(±120 kb)		0.0588	(3.38–21.92)	6.34 × 10^−8^	0.0094	(0.12–9.60)	0.96	0.0337	(1.98–10.91)	1.08 × 10^−4^
19	rs34282745	*ZNF154*	C/T	0.0382	4.34		0.0422	0.89		0.0388	2.49	
58214147		(within gene)		0.1471	(2.42–7.77)	7.40 × 10^−8^	0.0377	(0.30–2.67)	0.83	0.0914	(1.51–4.13)	2.44 × 10^−4^
14	rs737394	*ASPG*	C/A	0.1013	3.19		0.1000	0.73		0.1011	1.80	
104505922		(within gene)		0.2647	(2.02–5.04)	1.59 × 10^−7^	0.0755	(0.34–1.61)	0.44	0.1683	(1.23–2.63)	2.22 × 10^−3^
7	rs2214632	*ZNF804B*	A/G	0.178	2.86		0.1622	0.72		0.1758	1.56	
88513041		(within gene)		0.3824	(1.90–4.31)	1.80 × 10^−7^	0.1226	(0.38–1.36)	0.31	0.2500	(1.13–2.17)	6.49 × 10^−3^
11	rs7480329	*ADAMTS8*	A/G	0.0490	3.88		0.0400	0.46		0.0477	2.01	
130264278		(±10.5 kb)		0.1667	(2.24–6.72)	1.97 × 10^−7^	0.0189	(0.11–2.02)	0.29	0.0914	(1.22–3.30)	5.32 × 10^−3^
4	rs17658514	*SH3RF1*	T/C	0.0328	4.31		0.0378	0.48		0.0335	2.22	
170030472		(within gene)		0.1275	(2.32–8.00)	4.94 × 10^−7^	0.0185	(0.11–2.11)	0.32	0.0714	(1.27–3.88)	4.16 × 10^−3^
4	rs13118066	*SH3RF1*	C/A	0.0328	4.3		0.0378	0.49		0.0335	2.24	
170092033		(within gene)		0.1275	(2.32–7.99)	5.02 × 10^−7^	0.0189	(0.11–2.15)	0.34	0.0721	(1.28–3.92)	3.72 × 10^−3^
9	rs7026867	*ASTN2*	C/A	0.0083	7.48		0.0044	NA		0.0078	3.80	
120052359		(within gene)		0.0588	(2.98–18.80)	5.19 × 10^−7^	0		0.49	0.0289	(1.54–9.38)	1.82 × 10^−3^
17	rs16942475	*HOXB7*	C/T	0.0422	3.92		0.0622	0.73		0.0450	2.24	
46688371		(within gene)		0.1471	(2.20–6.98)	6.65 × 10^−7^	0.0463	(0.28–1.94)	0.53	0.0952	(1.37–3.65)	9.67 × 10^−4^
4	rs798752	*TMEM129*	A/C	0.0177	5.36		0.0200	0.47		0.0181	2.75	
1720312		(within gene)		0.0882	(2.56–11.24)	6.83 × 10^−7^	0.0094	(0.06–3.72)	0.46	0.0481	(1.38–5.46)	2.63 × 10^−3^
9	rs6560001	*DAPK1*	C/T	0.1445	2.83		0.1556	1.04		0.1460	1.85	
90169981		(within gene)		0.3235	(1.85–4.35)	6.92 × 10^−7^	0.1604	(0.58–1.85)	0.90	0.2404	(1.33–2.58)	2.35 × 10^−4^
12	rs17837141 *	*CLLU1*	C/A	0.0144	5.82		--	--		--	--	
92833965		(±9.2 kb)		0.0784	(2.65–12.77)	7.19 × 10^−7^	--	--	--	--	--	--

Abbreviations: A1, minor allele; A2, major allele; AF, allele frequency; Chr., chromosome; CI, confidence interval; NA, odds ratio cannot be estimated; OR, odds ratio. ^a^ Allele-based χ^2^-test (1 degree of freedom); * validation analysis of rs17837141 failed; chromosomal location is based on hg19. Replication analysis for the seven SNVs from NGS involved independent patients from ALL BFM 2000 and AIEOP BFM ALL 2009 (*n* = 45 cases; *n* = 45 controls) with available non-malignant DNA (most of which were part of the initial GWAS and replication cohorts). While for *ABCC4*, six variants demonstrated a tentative confirmatory behavior in replication analyses, leading to improved significance levels in combined analyses of initial discovery and replication sets, the *CFTR* SNV did not (Table 4). Five out of six variants localized on *ABCC4* had a *p*-value of 2.4 × 10^−2^ or less, although one has to acknowledge that four of these variants were highly linked to each other through LD. The most significant variant (rs4773862) had a *p*-value of 1.3 × 10^−2^, with 14 alleles present in the case group and 3 in controls.

**Table 4:** Top *ABCC4* and *CFTR* SNVs associated with AP identified through next-generation sequencing and replication analysis by Sanger sequencing.

Gene	Variant	A1/A2	Next-Generation Sequencing (Initial)			Sanger (Replication)			Combined Analysis (Initial + Replication)		
			Alleles_controls_ (A1/A2)	OR		Alleles_controls_ (A1/A2)	OR		Alleles_controls_ (A1/A2)	OR	
			Alleles_cases_(A1/A2)	(95% CI)	*p*-Value ^a^	Alleles_cases_ (A1/A2)	(95% CI)	*p*-Value ^a^	Alleles_cases_ (A1/A2)	(95% CI)	*p*-Value ^a^
*ABCC4*	rs34839857	GA/G	(7/81)	3.33		(8/64)	1.40		(15/145)	2.28	
			(21/73)	(1.34–8.29)	0.01	(11/63)	(0.53–3.70)	0.50	(32/136)	(1.18–4.39)	0.01
*ABCC4*	rs4773864	T/C	(2/90)	4.66		(1/78)	3.85		(3/165)	4.33	
			(9/87)	(0.98–22.16)	0.05	(4/78)	(0.42–35.21)	0.23	(13/165)	(1.21–15.49)	0.02
*ABCC4*	rs4773862	T/C	(2/92)	4.76		(1/81)	5.4		(3/173)	4.98	
			(9/87)	(1.00–22.64)	0.05	(5/75)	(0.62–47.29)	0.13	(14/162)	(1.41–17.66)	0.01
*ABCC4*	rs2027444	T/C	(2/92)	4.76		(1/81)	5.13		(3/173)	4.86	
			(9/87)	(1.00–22.64)	0.05	(5/79)	(0.59–44.87)	0.14	(14/166)	(1.37–17.23)	0.01
*ABCC4*	rs79230687	G/A	(2/92)	4.76		(1/81)	5.14		(3/173)	4.86	
			(9/87)	(1.00–22.64)	0.05	(5/79)	(0.59–44.87)	0.14	(14/166)	(1.37–17.23)	0.01
*CFTR*	rs62469434	A/G	(2/92)	5.95		(7/75)	0.54		(9/167)	1.69	
			(11/85)	(1.28–27.64)	0.02	(4/80)	(0.15–1.90)	0.34	(15/165)	(0.72–3.96)	0.23
*ABCC4*	rs2389226	C/A or T	(2/92)	4.76		(1/79)	5.13		(3/171)	4.87	
			(9/87)	(1.00–22.64)	0.05	(5/77)	(0.59–44.92)	0.14	(14/164)	(1.37–17.2)	0.01
*CFTR*	rs55831234	G/A	(0/90)	NA		(1/87)	1.93		(1/177)	4.89	
			(3/91)		0.25 *	(2(90)	(0.17–21.71)	0.59	(5/181)	(0.57–42.27)	0.15

Abbreviations: A1, minor allele; A2, major allele; CI, confidence interval; NA, odds ratio cannot be estimated; OR, odds ratio. ^a^ Unconditional logistic regression analysis; * Fisher’s exact test, as logistic regression analysis cannot be performed.

## Data Availability

Datasets of the current study are not publicly available but are available from the corresponding author on reasonable request.

## Supplementary Materials

The following are available online at https://www.mdpi.com/article/10.3390/jcm10214815/s1: Table S1. Treatment details of protocol AIEOP-BFM ALL 2000. Table S2. Additional information on top AP-associated SNV from GWAS and fine-mapping (NGS) analyses. Table S3. SNV within the *CPA2*, *PRSS1 and ULK2* genes previously identified by GWAS analyses and their association with AP in our cohort. Table S4. Genotype frequencies and association with risk of AP for SNV derived from fine-mapping by NGS analyses in the initial cohort. Table S5. Genotype frequencies and association with risk of AP for SNV derived from fine-mapping by NGS analyses in the replication cohort. Table S6: Genotype frequencies and association with risk of AP for SNV derived from fine-mapping by NGS analyses in the combined cohort (initial and replication). Table S7. Linkage disequilibrium of top *ABCC4* SNV from GWAS and fine-mapping analyses. Table S8. Linkage disequilibrium of *CFTR* SNV. Figure S1. Identification of individuals in the GWA scan of non-European ancestry. Figure S2. Quantile-quantile (Q-Q) plots showing observed vs. expected distribution of *p*-values for association of the GWAS-SNVs with Acute Pancreatitis (AP). Figure S3. Regional plots of the loci of the SNVs identified within the first approach of the GWAS of AP patients and controls (with the exception of the SNV located on the gene *ABCC4*, which is represented in Figure 1 in the article). Figure S4. Regional plots of the loci of the SNVs identified within the second approach of the GWAS of AP patients and controls (with the exception of the SNVs located on the genes *FGF10* and *ASPG*, which are represented in Figure 1 in the article).
