# Supplementary material for: Genetic Variation in ABCC4 and CFTR and Acute Pancreatitis during Treatment of Pediatric Acute Lymphoblastic Leukemia
Source: J Clin Med. 2021 Oct 20;10(21):4815. doi: 10.3390/jcm10214815 (PMC8584334; doi:10.3390/jcm10214815)
Supplement: Supplementary file 1 [file jcm-10-04815-s001.zip › jcm-1332118-supplementary.pdf]

SUPPORTING INFORMATION

Genetic variation in ABCC4 and CFTR and acute pancreatitis during treatment of pediatric acute lymphoblastic leukemia

Thies Bartram[1,2], Peter Schütte[2], Anja Möricke[1], Richard S. Houlston[3], Eva Ellinghaus[4], Martin Zimmermann[2], Anke Bergmann[5], Britt-Sabina Petersen[4], Norman Klein[2], Laura Hinze[2], Stefanie V. Junk[2], Michael Forster[4], Claus R. Bartram[6], Rolf Köhler[6], Andre Franke[4], Martin Schrappe[1], Christian Kratz[2], Gunnar Cario[1], Martin Stanulla[2*]

[1]Department of Pediatrics, University Hospital Schleswig-Holstein, Kiel, Germany
[2]Department of Pediatric Hematology and Oncology, Hannover Medical School, Hannover, Germany
[3]Division of Genetics and Epidemiology, Institute of Cancer Research, Surrey, UK
[4]Institute of Clinical Molecular Biology, Kiel University, Kiel, Germany
[5]Department of Human Genetics, Hannover Medical School, Hannover, Germany
[6]Department of Human Genetics, University Hospital Heidelberg, Heidelberg, Germany

* Correspondence: stanulla.martin@mh-hannover.de; Tel.: (+49-511-532 7978)

Table S1. Treatment details of protocol AIEOP-BFM ALL 2000.

Treatment phase/drug	Single or daily dose	Days of application per phase[a]
**Prephase**		
Prednisone (PO/IV)	60 mg/m²/d	1-7
Methotrexate (IT)	12 mg/dose[b]	1
**Induction**		
Protocol IA		
Prednisone/Prednisolone (PO/IV) *or*[c]	60 mg/m²/d	8-28[d]
Dexamethasone (PO/IV)	10 mg/m²/d	8-28[d]
Vincristine (IV)	1.5 mg/m²/dose (max 2 mg)	8, 15, 22, 29
Daunorubicin (PI over 1 h)	30 mg/m²/dose	8, 15, 22, 29
L-Asparaginase (PI over 1 h)[e]	5000 IU/m²/dose	12, 15, 18, 21, 24, 27, 30, 33
Methotrexate (IT)	12 mg/dose[b]	12, 33[f]
**Consolidation**		
Protocol IB		
Cyclophosphamide (PI over 1 h)	1000 mg/m²/dose	36, 64
Cytarabine (IV)	75 mg/m²/dose	38-41, 45-48, 52-55, 59-62
6-Mercaptopurine (PO)	60 mg/m²/d	36-63
Methotrexate (IT)	12 mg/dose[b]	45, 59
**Extra-Compartment Therapy (only SR/MR)**		
Protocol M:		
6-Mercaptopurine (PO)	25 mg/m²/d	1-56
Methotrexate (PI over 24 h)[g]	5000 mg/m²/dose	8, 22, 36, 50
Methotrexate (IT)	12 mg/dose[b]	8, 22, 36, 50
**Intensive Consolidation (only HR)**		
Element HR-1'		
Dexamethasone (PO/IV)	20 mg/m²/d	1-5
Vincristine (IV)	1.5 mg/m² (max 2 mg)	1, 6
Methotrexate (PI over 24 h)[g]	5000 mg/m²/dose	1
Cyclophosphamide (PI over 1 h)	200 mg/m²/dose	2-4 (5 doses, 12 h intervals)
Cytarabine (PI over 3 h)	2 g/m²/dose	5 (2 doses, 12 h interval)
L-Asparaginase (PI over 2 h)[e]	25,000 IU/m²/dose	6, 11
Methotrexate/Cytarabine/ Prednisolone (IT)	12/30/10 mg/dose[b]	1
Element HR-2'		
Dexamethasone (PO/IV)	20 mg/m²/d	1-5
Vindesine (IV)	3 mg/m²/dose (max 5 mg)	1, 6
Methotrexate (PI over 24 h)[g]	5000 mg/m²/dose	1
Ifosfamide (PI over 1 h)	800 mg/m²/dose	2-4 (5 doses, 12 h intervals)
Daunorubicin (PI over 24 h)	30 mg/m²/dose	5
L-Asparaginase (PI over 2 h)[e]	25,000 IU/m²/dose	6, 11
	12/30/10 mg/dose[b]	1[h]

Treatment phase/drug	Single or daily dose	Days of application per phase[a]
Methotrexate/Cytarabine/ Prednisolone (IT)		
Element HR-3′		
Dexamethasone (PO/IV)	20 mg/m^2/d	1-5
Cytarabine (PI over 3 h)	2 g/m^2/dose	1-2 (4 doses, 12 h intervals)
Etoposide (PI over 1 h)	100 mg/m^2/dose	3-5 (5 doses, 12 h intervals)
L-Asparaginase (PI over 2 h)[e]	25,000 IU/m^2/dose	6, 11
Methotrexate/Cytarabine/ Prednisolone (IT)	12/30/10 mg/dose[b]	5
**Reinduction**		
Protocol II		
Dexamethasone (PO/IV)	10 mg/m^2/d	1-21[d]
Vincristine (IV)	1.5 mg/m^2/dose (max 2 mg)	8, 15, 22, 29
Doxorubicin (PI over 1 h)	30 mg/m^2/dose	8, 15, 22, 29
L-Asparaginase (PI over 1 h)[e]	10,000 IU/m^2/dose	8, 11, 15, 18
Cyclophosphamide (PI over 1 h)	1000 mg/m^2/dose	36
Cytarabine (IV)	75 mg/m^2/dose	38-41, 45-48
6-Thioguanine (PO)	60 mg/m^2/d	36-49
Methotrexate (IT)	12 mg/dose[b]	45, 59[h]
Protocol III		
Dexamethasone (PO)	10 mg/m^2/d	1-14[d]
Vincristine (IV)	1.5 mg/m^2/dose (max 2 mg)	1, 8
Doxorubicin (PI over 1 h)	30 mg/m^2/dose	1, 8
L-Asparaginase (PI over 1 h)[e]	10,000 IU/m^2/dose	1, 4, 8, 11
Cyclophosphamide (PI over 1 h)	500 mg/m^2/dose	15
Cytarabine (IV)	75 mg/m^2/dose	17-20, 24-27
6-Thioguanine (PO)	60 mg/m^2/d	15-28
Methotrexate (IT)	12 mg/dose[b]	17, 24[h]
**Interim Maintenance**		
Methotrexate (PO)	20 mg/m^2/dose	once a week
6-Mercaptopurine (PO)	50 mg/m^2/d	daily
**Maintenance**[i]		
Methotrexate (PO)	20 mg/m^2/dose[j]	once a week
6-Mercaptopurine (PO)	50 mg/m^2/d[j]	daily
Cranial irradiadion	12 Gy/18 Gy/24 Gy	

[a]Adjustments of time schedule were allowed if clinical condition and bone marrow recovery were inadequate.
[b]Doses of IT drugs were adjusted for children <3 years of age.
[c]Randomization
[d]Steroids were tapered over 9 additional days.
[e] L-Asparaginase dose was not modified in case of asymptomatically elevated amylase and/or lipase levels. If there was confirmed pancreatitis, L-Asparaginase therapy had to be discontinued. As a matter of principle it was not recommended to continue L-Asparaginase therapy (including with other preparations, e.g. Erwinia Asparaginase) after acute pancreatitis. In case of very mild forms of pancreatitis (no correlation in image morphology, improvement after only a few days with conservative treatment), reinstatement of L-Asparaginase may have been considered following consultation with the study coordination center.

[f]Additional IT therapy on day 18 and 27 was administered to patients with CNS status CNS3 and CNS2 or TLP+.
[g]A loading dose of 10% was infused over 30 min, the remaining 90% over 23.5 h. Leucovorin rescue was given at hour 42, 48, and 54 (each 15 mg/m^2). Doses of leucovorin rescue were adjusted, if MTX levels were > 1.0 μmol/l at hour 42 or later. If the MTX level at hour 54 was > 0.25 μmol/l, rescue was continued at six-hour intervals until MTX levels were ≤ 0.25 μmol/l.
[h]Patients with CNS status CNS 3 received additional IT therapy on day 5 in element HR-2' (only BFM), on day 1 and 18 in Protocol II and on day 1 in Protocol III.
[i]Maintenance was given from the end of intensive chemotherapy until 104 weeks after diagnosis.
[j]Doses were adjusted to WBC (target range 2.0-3.0 x10^9/L).
PO indicates orally; IV, intravenous push; PI, intravenous infusion; IT, intrathecally.

Table S2. Additional information on top AP-associated SNV from GWAS and fine-mapping (NGS) analyses.

Cohort	Chr	Position (bp)	dbSNP ID	Nearby genes	Category	Variant position	Comment
GWAS	13	95790353	rs4148513	*ABCC4*	SNV	intronic	intron 13
GWAS	7	85465201	rs17160216	*SEMA3D*	SNV	intergenic	--
GWAS	15	36372995	rs698457	*C15orf41*	SNV	intergenic	--
GWAS	7	106850181	rs6963190	*COG5*	SNV	exonic	exone 21, UTR3'
GWAS	7	116857547	rs7804397	*ST7*	SNV	intronic	intron 13
GWAS	19	18942559	rs2238652	*UPF1*	SNV	intergenic	185 bp from 5'
GWAS	13	95818288	rs4148500	*ABCC4*	SNV	intronic	intron 17
GWAS	5	44005497	rs6858970	*FGF10*	SNV	intergenic	--
GWAS	1	118847717	rs12402476	*SPAG17*	SNV	intergenic	--
GWAS	19	58214147	rs34282745	*ZNF154*	SNV	exonic	exone 3, missense (H->R)
GWAS	14	104565922	rs737394	*ASPG*	SNV	intronic	intron 6
GWAS	7	88513041	rs2214632	*ZNF804B*	SNV	intronic	intron 1
GWAS	11	130264278	rs7480329	*ADAMTS8*	SNV	intergenic	--
GWAS	4	170030472	rs17658514	*SH3RF1*	SNV	intronic	intron 2
GWAS	4	170092033	rs13118066	*SH3RF1*	SNV	intronic	intron 10
GWAS	9	120052359	rs7026867	*ASTN2*	SNV	intronic	intron 20
GWAS	17	46688371	rs16942475	*HOXB7*	SNV	exonic	exone 1, UTR5'
GWAS	4	1720312	rs798752	*TMEM129*	SNV	exonic	exone 2, missense (L->I)
GWAS	9	90170231	rs6560001	*DAPK1*	SNV	intronic	intron 2
GWAS	12	92833965	rs17837141	*CLLU1*	SNV	intergenic	--
NGS	13	95680323	rs34839857	*ABCC4*	Insertion	intronic	intron 30
NGS	13	95925010	rs4773864	*ABCC4*	SNV	intronic	intron 1
NGS	13	95910230	rs4773862	*ABCC4*	SNV	intronic	intron 1
NGS	13	95919634	rs2027444	*ABCC4*	SNV	intronic	intron 1
NGS	13	95922306	rs79230687	*ABCC4*	SNV	intronic	intron 1
NGS	7	117140634	rs62469434	*CFTR*	SNV	intronic	intron 1
NGS	13	95910321	rs2389226	*ABCC4*	SNV	intronic	intron 1
NGS	7	117308456	rs55831234	*CFTR*	SNV	exonic	exone 27, UTR3'

Abbreviations: Chr, chromosome; bp, base pair; dbSNP, Single Nucleotide Polymorphism database; UTR, untranslated region; H, Histidine; R, Arginine; L, Leucine; I, Isoleucine

Table S3. SNV within the *CPA2, PRSS1 and ULK2* genes previously identified by GWAS analyses and their association with AP in our cohort.

Gene	Chr:position (bp)	dbSNP ID	Location	Function	A1/A2	AF_{A1} contr.	AF_{A1} cases	P value[a]	OR	95% CI
CPA2	7:129912602	rs2242337	intronic		G/A	0.2954	0.2451	0.27	0.77	0.49 - 1.23
	7:129912991				-/G	0	0	NA	NA	
	7:129916030	rs9641869	intronic		G/A	0.1525	0.1569	0.9	1.03	0.60 - 1.78
	7:129916501				-/A	0	0	NA	NA	
	7:129916515	rs17590	exonic	silent	T/C	0.4009	0.4118	0.83	1.05	0.70 - 1.56
	7:129916532				-/T	0	0	NA	NA	
	7:129918947	rs10244258	intronic		-/C	0	0	NA	NA	
	7:129920239	rs10215911	intronic		T/C	0.4009	0.4118	0.83	1.05	0.70 - 1.56
	7:129923765	rs2178158	intronic		A/G	0.1016	0.0882	0.66	0.86	0.43 - 1.72
	7:129925510	rs10242953	intronic		-/T	0	0	NA	NA	
	7:129927778	rs6467293	intronic		A/G	0.1381	0.1275	0.76	0.91	0.50 - 1.65
PRSS1	7:142136502	rs10273639			T/C	0.4423	0.3431	0.05	0,66	0.43 - 1.00
ULK2	17:19674364	rs11658829	intronic		A/C	0.0004	0	0.85	0	
	17:19675013	rs9900160	intronic		A/G	0.0007	0	0.79	0	
	17:19675104	rs205111	intronic		A/G	0.0321	0.0098	0.20	0.3	0.04 - 2.17
	17:19675547	rs9895806	intronic		T/C	0	0	NA	NA	
	17:19675588	rs205110	intronic		G/A	0.0331	0.0098	0.19	0.29	0.04 - 2.09
	17:19675775	rs17720618	intronic		C/G	0.1190	0.1176	0.97	0.99	0.53 - 1.82
	17:19676673	rs9914674	intronic		-/A	0	0	NA	NA	
	17:19676691	rs118943	intronic		T/C	0.0331	0.0098	0.19	0.29	0.04 - 2.09
	17:19677749	rs115326	intronic		G/C	0.0332	0.0098	0.19	0.29	0.04 - 2.09
	17:19678308	rs205108	intronic		T/G	0.0375	0.0196	0.35	0.51	0.13 - 2.11
	17:19678475	rs205107	intronic		C/T	0.0332	0.0098	0.19	0.29	0.04 - 2.09
	17:19679721				-/C	0	0	NA	NA	
	17:19684290	rs1662214	intronic		-/G	0	0	NA	NA	

17:19684364				-/G	0	0	NA	NA	
17:19692809	rs11656704	intronic		A/G	0.2383	0.2059	0.45	0.83	0.51 - 1.35
17:19697976	rs17794370	intronic		A/G	0.2752	0.2353	0.37	0.81	0.51 - 1.30
17:19699121	rs157386	intronic		T/C	0.1348	0.1700	0.31	1.31	0.77 - 2.24
17:19713740	rs150122	exonic	missense	G/A	0.0335	0.0098	0.19	0.29	0.04 - 2.07
17:19714206	rs157407	intronic		C/T	0.0513	0.0294	0.32	0.56	0.18 - 1.79
17:19720838	rs16960454	intronic		C/T	0.2628	0.3137	0.25	1.28	0.84 - 1.97
17:19724155	rs157395	intronic		G/A	0.0335	0.0098	0.17	0.29	0.04 - 2.07
17:19728077	rs9898591	intronic		C/T	0	0.0098	1.85×10^{-7}	NA	

Abbreviations: A1, minor allele; A2, major allele; AF, allele frequency; Chr, chromosome; CI, confidence interval; NA, not analyzed; OR, odds ratio.

[a]allele-based χ^2–test (one degree of freedom); chromosomal location is based on hg19.

Table S4. Genotype frequencies and association with risk of AP for SNV derived from fine-mapping by NGS analyses in the initial cohort.

Gene	Variant	Genotype	Cases (%)	Controls (%)	OR (95% CI)	*P* value[a]
ABCC4	rs34839857	G_G_	32 (68.1)	38 (86.4)	1.00 (referent)	
		G_GA	9 (19.1)	5 (11.4)	2.14 (0.65-7.03)	0.211
		GAGA	6 (12.8)	1 (2.3)	7.13 (0.82-62.32)	0.076
ABCC4	rs4773864	CC	39 (81.2)	44 (95.7)	1.00 (referent)	
		CT	9 (18.7)	2 (4.3)	5.08 (1.03-24.94)	0.045
		TT	0	0		
ABCC4	rs4773862	CC	39 (81.2)	45 (95.7)	1.00 (referent)	
		CT	9 (18.7)	2 (4.3)	5.19 (1.06-25.49)	0.042
		TT	0	0		
ABCC4	rs2027444	CC	39 (81.2)	45 (95.7)	1.00 (referent)	
		CT	9 (18.7)	2 (4.3)	5.19 (1.06-25.49)	0.042
		TT	0	0		
ABCC4	rs79230687	AA	39 (81.2)	45 (95.7)	1.00 (referent)	
		AG	9 (18.7)	2 (4.3)	5.19 (1.06-25.49)	0.042
		GG	0	0		
CFTR	rs62469434	GG	38 (79.2)	45 (95.7)	1.00 (referent)	
		GA	9 (18.7)	2 (4.3)	5.33(1.09-26.18)	0.039
		AA	1 (2.1)	0	--	1.00
ABCC4	rs2389226	TT/AA	39 (81.2)	45 (95.7)	1.00 (referent)	
		CT/CA	9 (18.7)	2 (4.3)	5.19 (1.06-25.49)	0.042
		CC	0	0	--	

Abbreviations: OR, odds ratio; CI, confidence interval

[a]unconditional logistic regression analysis

Table S5. Genotype frequencies and association with risk of AP for SNV derived from fine-mapping by NGS analyses in the replication cohort.

Gene	Variant	Genotype	Cases (%)	Controls (%)	OR (95% CI)	*P* value[a]
ABCC4	rs34839857	G_G_	26 (70.3)	29 (80.6)	1.00 (referent)	
		G_GA	11 (29.7)	6 (16.7)	2.05 (0.66-6.31)	0.213
		GAGA	0	1 (2.7)		1.00
ABCC4	rs4773864	CC	37 (90.2)	37 (97.4)	1.00 (referent)	
		CT	4 (9.8)	1 (2.6)	4.00 (0.43-37.51)	0.225
		TT	0	0		
ABCC4	rs4773862	CC	35 (87.5)	40 (97.6)	1.00 (referent)	
		CT	5 (12.5)	1 (2.4)	5.71 (0.64-51.29)	0.120
		TT	0	0		
ABCC4	rs2027444	CC	37 (88.1)	40 (97.6)	1.00 (referent)	
		CT	5 (11.9)	1 (2.4)	5.41 (0.60-48.45)	0.132
		TT	0	0		
ABCC4	rs79230687	AA	37 (88.1)	40 (97.6)	1.00 (referent)	
		AG	5 (11.9)	1 (2.4)	5.41 (0.60-48.45)	0.132
		GG	0	0		
CFTR	rs62469434	GG	38 (90.5)	34 (82.9)	1.00 (referent)	
		GA	4 (9.5)	7 (17.1)	0.51 (0.14-1.90)	0.317
		AA	0	0	--	
ABCC4	rs2389226	TT/AA	36 (87.8)	39 (97.5)	1.00 (referent)	
		CT/CA	5 (12.2)	1 (2.5)	5.42 (0.60-48.61)	0.131
		CC	0	0	--	

Abbreviations: OR, odds ratio; CI, confidence interval
[a]unconditional logistic regression analysis

Table S6: Genotype frequencies and association with risk of AP for SNV derived from fine-mapping by NGS analyses in the combined cohort (initial and replication).

Gene	Variant	Genotype	Cases (%)	Controls (%)	OR (95% CI)	*P* value[a]
ABCC4	rs34839857	G_G_	58 (69)	67 (83.8)	1.00 (referent)	
		G_GA	20 (23.8)	11 (13.8)	2.1 (0.93-4.75)	0.070
		GAGA	6 (7.1)	2 (2.5)	3.47 (0.67-17.84)	0.137
ABCC4	rs4773864	CC	76 (85.4)	81 (96.4)	1.00 (referent)	
		CT	13 (14.6)	3 (3.6)	4.62 (1.27-16.84)	0.020
		TT	0	0		
ABCC4	rs4773862	CC	74 (84.1)	85 (96.6)	1.00 (referent)	
		CT	14 (15.9)	3 (3.4)	5.36 (1.48-19.38)	0.010
		TT	0	0		
ABCC4	rs2027444	CC	76 (84.4)	85 (96.6)	1.00 (referent)	
		CT	14 (15.6)	3 (3.4)	5.22 (1.44-18.86)	0.012
		TT	0	0		
ABCC4	rs79230687	AA	76 (84.4)	85 (96.6)	1.00 (referent)	
		AG	14 (15.6)	3 (3.4)	5.22 (1.44-18.86)	0.012
		GG	0	0		
CFTR	rs62469434	GG	76 (84.4)	79 (89.8)	1.00 (referent)	
		GA	13 (14.4)	9 (10.2)	1.5 (0.61-3.72)	0.379
		AA	1 (1.1)	0	--	1.00
ABCC4	rs2389226	TT/AA	75 (84.3)	84 (96.6)	1.00 (referent)	
		CT/CA	14 (15.7)	3 (3.4)	5.23 (1.45-18.90)	0.012
		CC	0	0	--	

Abbreviations: OR, odds ratio; CI, confidence interval

[a]unconditional logistic regression analysis

Table S7. Linkage disequilibrium of top *ABCC4* SNV from GWAS and fine-mapping analyses.

dbSNP ID	rs34839857		rs4773864		rs4773862		rs2027444		rs79230687		rs2389226		rs4148513*	
	r^2	D'	r^2	D'	r^2	D'	r^2	D'	r^2	D'	r^2	D'	r^2	D'
rs34839857	x	x	0.002	0.409	0.002	0.403	0.002	0.403	0.002	0.403	0.004	0.457	0.026	0.527
rs4773864	0.002	0.409	x	x	1	1	1	1	1	1	0.668	1	0.016	0.249
rs4773862	0.002	0.403	1	1	x	x	1	1	1	1	0.669	1	0.016	0.25
rs2027444	0.002	0.403	1	1	1	1	x	x	1	1	0.669	1	0.016	0.25
rs79230687	0.002	0.403	1	1	1	1	1	1	x	x	0.669	1	0.016	0.25
rs2389226	0.004	0.457	0.668	1	0.669	1	0.669	1	0.669	1	x	x	0.013	0.273
rs4148513*	0.026	0.527	0.016	0.249	0.016	0.25	0.016	0.25	0.016	0.25	0.013	0.273	x	x

*= Top candidate SNV from GWAS

Table S8. Linkage disequilibrium of *CFTR* SNV.

dbSNP ID	rs62469434		rs55831234	
	r^2	D'	r^2	D'
rs62469434	x	x	0.011	0.228
rs55831234	0.011	0.228	x	x

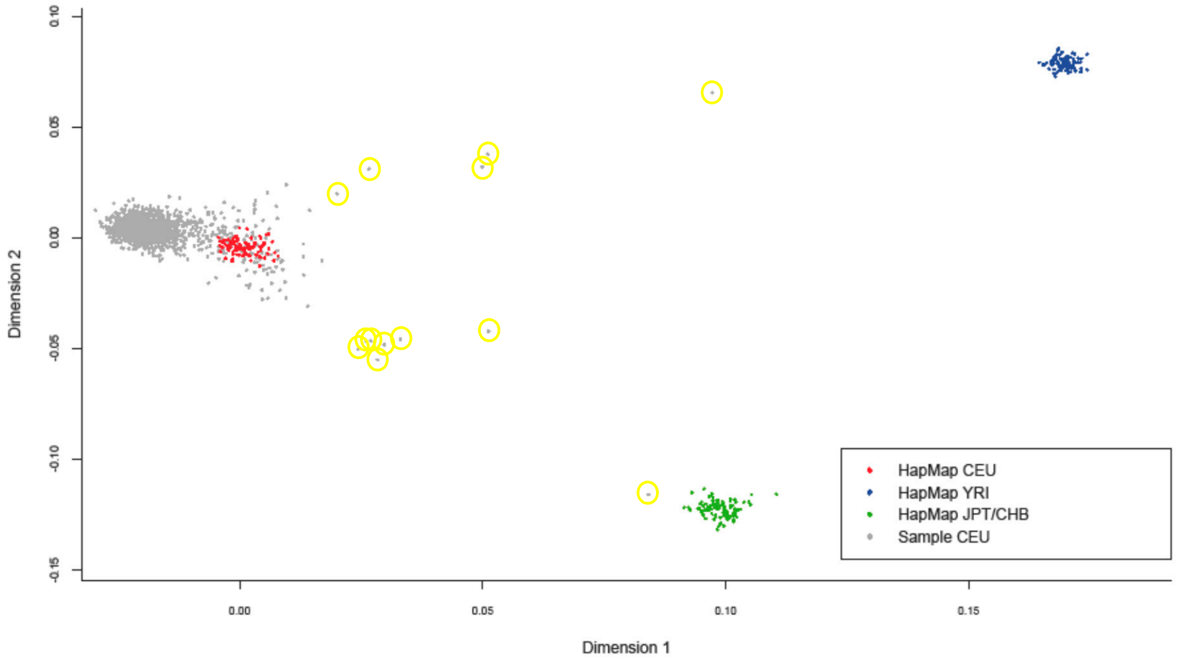

Figure S1. Identification of individuals in the GWA scan of non-European ancestry. The multidimensional scaling plot (MDS) shows genuine European ancestry for the cleaned GWAS cohort, which was plotted with the three distinct HapMap sample populations (see legend box in the plot) for the first two principle components. Thirteen samples showed evidence of non-European ancestry (see yellow circles in the plot) and were therefore removed.

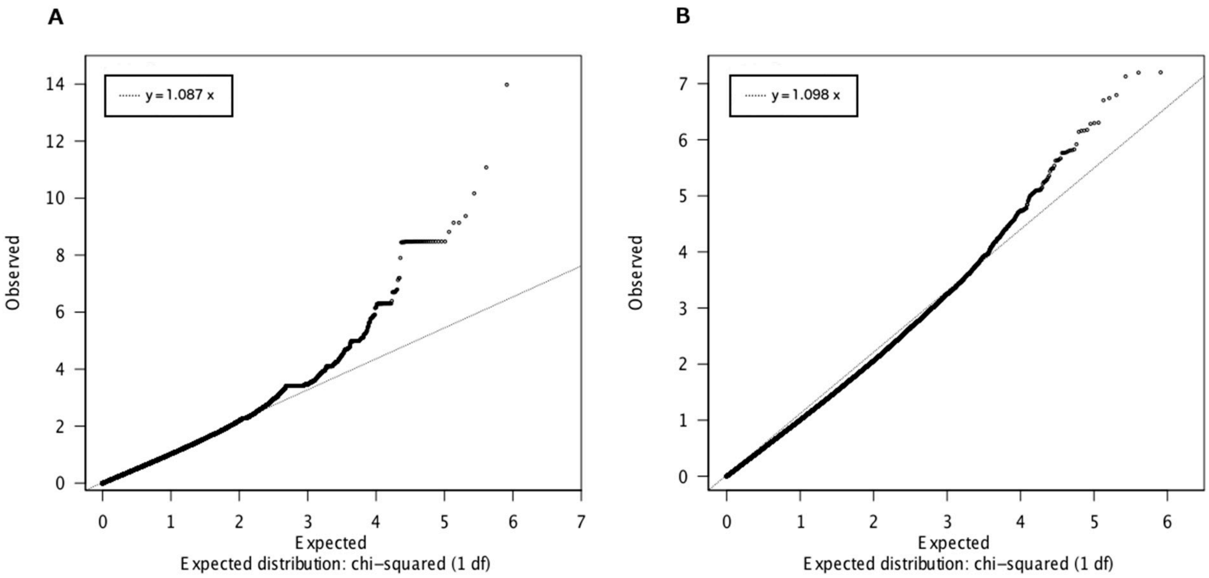

Figure S2. Quantile-quantile (Q-Q) plots showing observed vs. expected distribution of P-values for association of the GWAS-SNVs with Acute Pancreatitis (AP). For this analysis only genotyped SNVs that passed quality control were included.

(A) Q-Q-plot of all SNVs included in approach 1 of the GWAS study. The plot was calculated under exclusion of all SNVs within the extended MHC region (Chr. 6, 29-33 Mb: n=10,815 SNVs in interval), leaving 817,184 SNVs for plotting. The genomic inflation factor, based on the median chi-squared (λ=1.09) indicated no or minimal undetected population stratification or cryptic relatedness.

(B) Q-Q-plot of all SNVs included in approach 2 of the GWAS study. The plot was calculated under exclusion of all SNVs within the extended MHC region (Chr. 6, 29-33 Mb: n=9,604 SNVs in interval), leaving 809,126 SNVs for plotting. The genomic inflation factor, based on the median chi-squared (λ=1.10) indicated no or minimal undetected population stratification or cryptic relatedness.

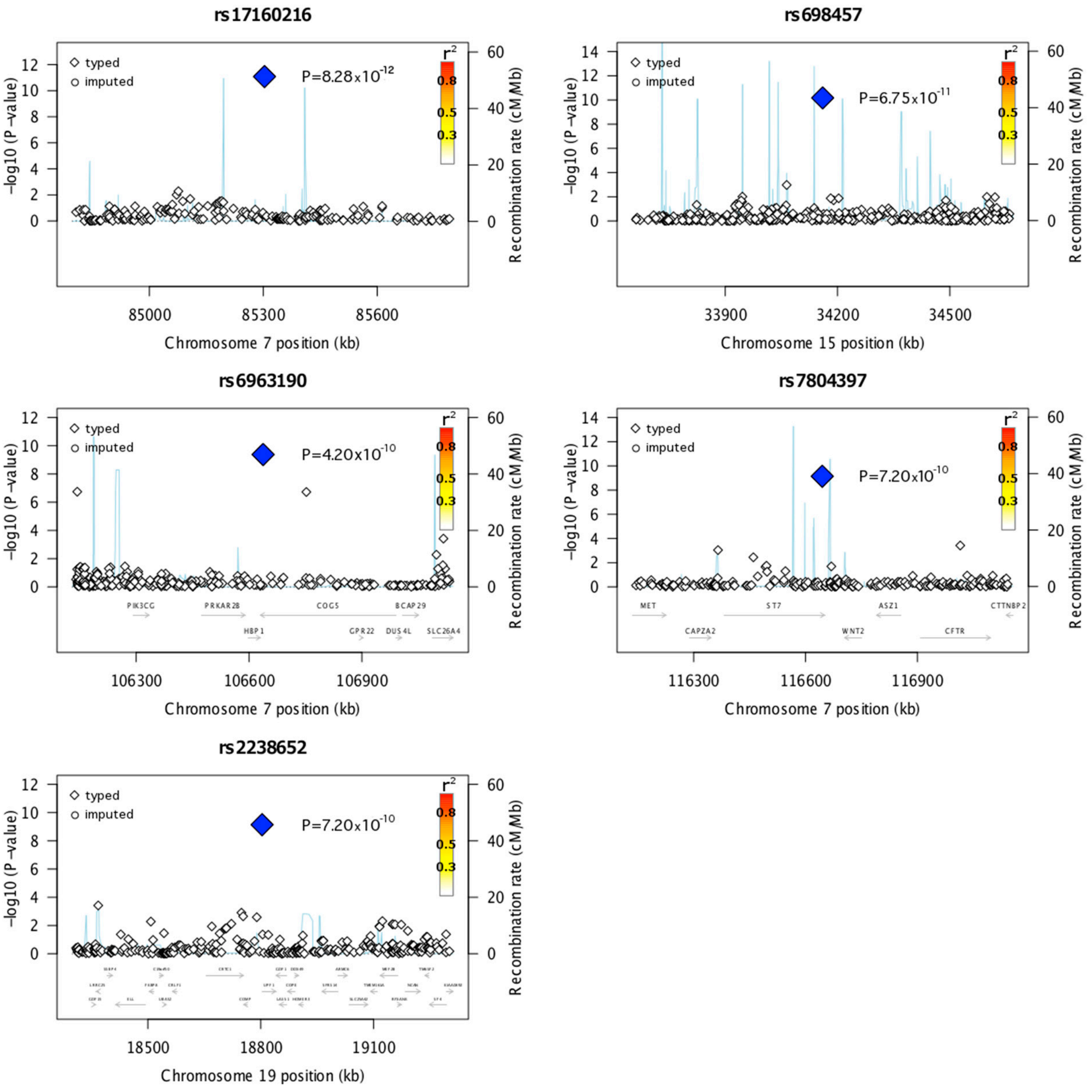

Figure S3. Regional plots of the loci of the SNVs identified within the first approach of the GWAS of AP patients and controls (with exception of the SNV located at the gene *ABCC4*, which is represented in Figure 1 in the article). The chromosome position (x-axis) of SNVs near the candidate SNV is plotted against their association with AP (y-axis). The degree of linkage disequilibrium (r^2) for each SNV located in the vicinity of the candidate SNV is color-coded. Recombination activity (in centimorgans (cM) per Mb) is depicted by a blue line. Positions are given as NCBI's build coordinates.

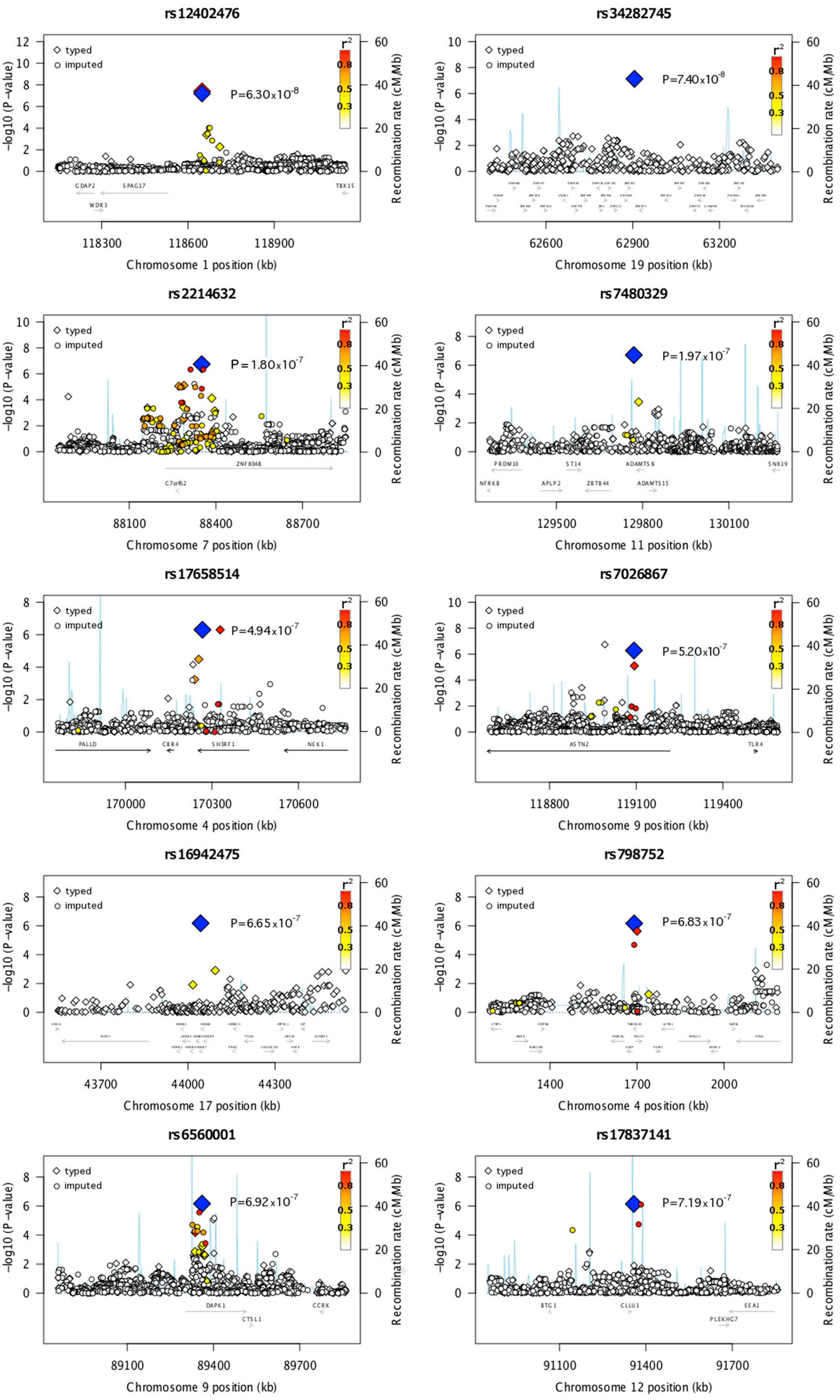
rs12402476
P=6.30x10-8
Chromosome 1 position (kb)
GDAP2
SPAG17
WDR3
TBX15
rs34282745
P=7.40x10-8
Chromosome 19 position (kb)
rs2214632
P=1.80x10-7
Chromosome 7 position (kb)
ZNF804B
C7orf62
rs7480329
P=1.97x10-7
Chromosome 11 position (kb)
PRDM10
ST14
ADAMTS8
SNX19
NFRKB
APLP2
ZBTB44
ADAMTS15
rs17658514
P=4.94x10-7
Chromosome 4 position (kb)
PALLD
CBR4
SH3RF1
NEK1
rs7026867
P=5.20x10-7
Chromosome 9 position (kb)
ASTN2
TLR4
rs16942475
P=6.65x10-7
Chromosome 17 position (kb)
rs798752
P=6.83x10-7
Chromosome 4 position (kb)
rs6560001
P=6.92x10-7
Chromosome 9 position (kb)
DAPK1
CTSL1
CCRK
rs17837141
P=7.19x10-7
Chromosome 12 position (kb)
BTG1
CLLU1
EEA1
PLEKHG7
typed
imputed
-log10 (P-value)
Recombination rate (cM/Mb)
r2

Figure S4. Regional plots of the loci of the SNVs identified within the second approach of the GWAS of AP patients and controls (with exception of the SNVs located at the genes *FGF10* and *ASPG*, which are represented in Figure 1 in the article).The chromosome position (x-axis) of SNVs near the candidate SNV is plotted against their association with AP (y-axis). The degree of linkage disequilibrium (r2) for each SNV located in the vicinity of the candidate SNV is color-coded. Recombination activity (in centimorgans (cM) per Mb) is depicted by a blue line. Positions are given as NCBI's build coordinates.
